# Assessment of electrical impedance endotomography for hardware specification

**DOI:** 10.2349/biij.2.2.e24

**Published:** 2006-04-01

**Authors:** J Jossinet, A Fournier-Desseux, A Matias

**Affiliations:** National Institute for Health and Medical Research, INSERM U556, Lyon, France

**Keywords:** Electrical impedance tomography, drive pattern, sensitivity, noise, hardware design, urethral probe, prostate

## Abstract

**Purpose:**

The purpose of the study is the quantitative assessment of Electrical Impedance Endotomography (EIE) for the specification of hardware systems. EIE is a modality of Electrical Impedance Tomography (EIT) where the electrodes are located on a probe placed in the middle of the region of interest. The absence of material boundary to the explored volume and the decrease in sensitivity away from the probe requires specific study.

**Material and methods:**

The method is the derivation of the equation linking explored medium’s conductivity, the sensitivity distribution of the electrode patterns used for data collection and measuring system’s noise and bandwidth. The assessment of EIE was achieved by means of simulations based on realistic data of conductivity and noise level.

**Results:**

The derived equation enabled the estimation of the current needed under realistic operating conditions corresponding to prostate imaging. The generalisation to other organs is straightforward. The image reconstructed from the simulated data and from bench experiments were in agreement and showed that the two selected drive patterns, fan3 and adjacent, gave images of similar quality in absence of noise and that adjacent drive requires significantly higher measurement current.

**Conclusion:**

The study confirmed the feasibility of EIE with achievable hardware specifications. The derived equation enabled the determination of design parameters for the specification of hardware systems corresponding to any given application. The study also showed that EIE is more appropriate for tissue characterisation than for high speed imaging.

## INTRODUCTION

Electrical Impedance Tomography (EIT) produces images of a body from impedance data collected using surface electrodes. The advantages of this method in medical applications include harmlessness, ease-of-use, high time resolution and specificity for tissue characterisation [1-4]. The direct problem obeys the second order differential equation Ñ·(σ Ñu) = 0 with Neumann/Dirichlet mixed boundary conditions. The associated inverse problem is ill-posed [5]. The small number of measurements limits the spatial resolution of the reconstructed images. The reduction of sensitivity and resolution from periphery towards the centre [6] makes EIT not well suited for the imaging of small and deep-located organs. The nature of the governing equation and the low number of possible measurements with a workable number of electrodes prevents significant improvement of spatial resolution.

**Figure 1:**
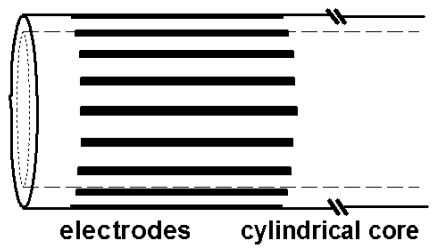
Sketch of the tip of a 16-electrode probe for EIE. The insulating core may consist of a cylindrical tube enabling to pass electrode leads.

In EIE, the electrodes are located on a probe placed in the middle of the region of interest for local measurements. An EIE probe consists of multiple linear electrodes regularly spaced on the outer surface of an insulating core (Figure 1). EIE was developed for prostate imaging aimed at the evaluation of cancer treatment by therapeutic ultrasound [7-9]. In the proposed application, the impedance method is expected to compensate for the lack of specificity of ultrasonic imaging in cancer detection and the low acoustic contrast observed in the prostate between normal tissue and tissue treated by ultrasound [10-12]. This approach has been supported by the known significant conductivity differences between cancerous and normal tissue observed in various organs [13-18], including human prostate [19,20], and by recent studies reporting significant conductivity changes in tissue exposed to ultrasound energy [21,22]. More generally, EIE can potentially address a range of interstitial and intracanular measurements such as in oesophageal and vascular studies.

The method, however, does not aim to compete with the radiological methods of prostate imaging. The objective is to derive a complementary technique for use in conjunction with ultrasound techniques. The objective is to exploit the specificity of impedance measurements to improve the characterisation of tissue before and after treatments with therapeutic ultrasound.

EIE obeys the same governing equation as EIT but with different boundary conditions. The boundary profile is unknown and variable in EIT according to the morphology of the examined region and inter-patient variability while the surface bearing the electrodes is known by construction in EIE. It is obvious that the volume actually sensed by the probe is finite although there is no material boundary around the probe. The limit is the distance beyond which noise overrides the contributions of distant points.

The determination of the computational domain is a key problem in EIE. The absence of tangible limit for the domain sensed by the EIE probe is one major difference compared with EIT. Therefore, the notions formerly investigated in EIT are still relevant in EIE, due to the same governing equation, but need to be revisited. For quantitative studies, the geometry of an EIE probe suggested the use of a model with axial symmetry. In this 2D model, an infinitely long cylinder represents the core of the probe and infinitely long lines regularly spaced at the outer surface of this cylinder represent the electrodes. This model enabled the derivation of analytical equations for current density, field and potential created by EIE electrodes in a medium of homogeneous conductivity.

According to the model, the field of a single electrode is proportional to 1/d, where d is the distance to the centre of the probe. Hence, the field of a pair of electrodes tends to vary as 1/d^2^ for large distances to the probe. The consequence is a rapid fall-off in sensitivity of the order of 1/d^4^. The measurements in a semi-infinite medium present a certain similarity with the case of the rosette array of surface electrodes used in EIT for monitoring gastric function [23]. In both cases, the electrodes are grouped together, do not encircle the region of interest, and explore a semi-infinite medium. The measurements carried out with the rosette support the feasibility of EIE measurements.

**Figure 11:**
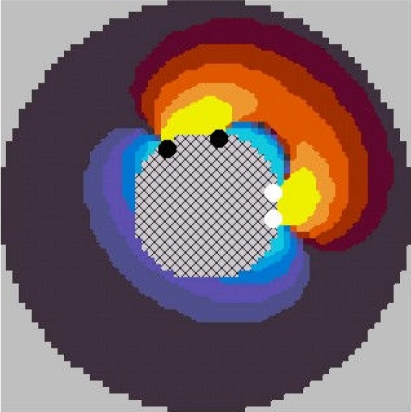
This animation displays successively the sensitivity maps of the 49 basic patterns from which any electrode pattern can be derived by symmetry and rotation. This animation does not display any time varying process. The purpose is to illustrate the influence of drive pattern on the sensitivity distribution around an EIE probe comprising 16 electrodes. The red colours show positive values of sensitivity and blue colours to negative ones. The highest magnitude values are near the electrode. The change is 10 dB from one colour level to the next. The central, background colour corresponds to low absolute values of sensitivity, either positive or negative. As there are 11 levels, the background corresponds to sensitivity smaller than 50 dB compared to the maximum. The maps were drawn varying the current injection pair of electrodes and keeping constant the sensing pair. The radius of the mapped zone is 3 times the radius of the probe.

It was found that for a total number of 16 electrodes, the possible 4-electrode patterns using an adjacent pair of voltage electrodes can be sorted into 49 basic patterns from which any pattern can be obtained by symmetry and rotation. The association of the model with the lead field theory enabled the calculations of sensitivity maps for the basic 49 patterns. The study of these maps showed that the extension of sensitivity increases with the angular spacing of source electrodes. These maps are shown in the form of an animation (Figure 11). The drive pattern giving the largest sensitivity range was selected based on these sensitivity maps.

The purpose of this study is the quantitative assessment of EIE as a whole including medium, electrodes and instrumentation. This differs from the previous studies, which were limited to the comparison of drive patterns to determine the widest sensitivity range. The novelty of the present study is to encompass all the components involved in EIE data collection. This was achieved by the derivation of an equation linking measurement, noise, magnitude of injected current, electrode sensitivity distribution, medium conductivity and conductivity contrast to be observed. The inclusion of noise enabled the calculation of the volume actually sensed by the probe. The study is supported by the comparison of adjacent and fan3 drive patterns using calculated data and images reconstructed from computer and experimental data. Although particular attention was given to prostate imaging, the study has been intended to enable generalisation to other applications and the design of hardware systems.

## DEFINITIONS

### Electrode patterns

In this study, the measurements were carried out according to the 4-electrode technique with bipolar current patterns and differential voltage sensing with all four electrodes located on the probe. The sensing pair always consists of adjacent electrodes for hardware reasons including reduction of common mode signal. The number of patterns with adjacent voltage electrodes is N_T_ = (N_E_-3) (N_E_-2)N_E_/2, where N_E_ is the number of electrodes on the probe. The maximum number of linearly independent patterns is (N_E_-3)N_E_/2, as this was formerly demonstrated in EIT. In this study, it was found convenient to consider that the set of 4-electrode patterns used for data acquisition consists of the N_E_ angular duplications of a basic set consisting of patterns comprising a given pair of voltage electrodes (pair arbitrarily denoted {0,1} in this study) associated to different pairs of current electrodes.

### Reconstruction mesh

**Figure 2:**
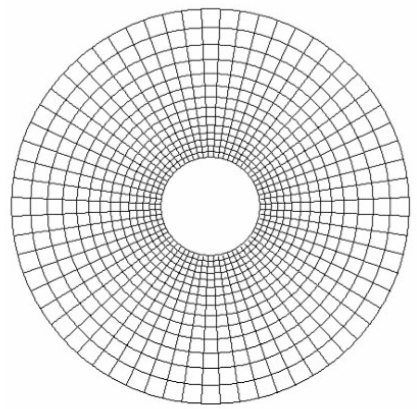
Reconstruction mesh used in this study. The central area corresponds to the insulating core of the probe. There are 14 layers and 64 angular sectors forming 896 trapezoidal pixels of constant profile but varying dimensions.

The reconstruction mesh consisted of N_L_ concentric layers of N_A_ trapezoidal pixels. The outer radius of the mesh was denoted R_max_. The vertices of a pixel were located on two circles of radii r_n-1_ and r_n_, with r_n-1_ < r_n_, r_0_=1 and r_NL_ = R_max_. The number of layers was N_C_ = 14, the number of angular sectors was N_A_ = 64. Hence, the number of pixels was N_pix_ = 896. The mesh was designed, so that pixel dimensions were proportional to the distance from the origin (Figure 2). This was achieved in the setting of the radial increment equal to the length of the circular arc passing by the centre of the pixel. This condition can be written under the form of (1):

(1) $$r_{n}=r_{n-1}\left(N_{A}+\pi \right)/\left(N_{A}-\pi \right)$$

In this mesha design, the resolution is better for pixels of higher sensitivity while the increase in pixel size with distance tends to compensate for the sensitivity decrease. The radius of the reconstructed domain was chosen according to the size of the domain explored by the probe. If the reconstruction domain is too small, significant elements would be ignored and attributed erroneously to pixels located inside the mesh. If the reconstruction domain is too wide, it would encompass points with negligible contributions. In this study, the reconstruction domain was determined by considering the diameters of urethral probes used in urological practice and the size of the prostate. This 3-cm high organ is approximately conical in shape. It presents a base, an anterior, a posterior and two lateral surfaces. The base applied to the inferior surface of the bladder and the apex is directed downwards. The prostate is about 4×2 cm^2^ at the base (2 cm in antero-posterior diameter). From these dimensions, the value of R_max_ chosen was equal to 4 times the radius of the probe. This justified the use of 14-layer mesh.

### Sensitivity

Sensitivity is a general concept to quantify the change in the measured signal when the conductivity within a given element, ∆τ, changes by ∆σ. The computation of sensitivity normally requires the resolution of the governing equation knowing the original and the perturbed distribution of conductivity in the medium. For small perturbations, the lead field theory yields a linear approximation that has been widely used in impedance imaging. This theory has enabled the derivation of a general expression of change, ∆Z_x_, in the measured impedance, Z_x_, due to the conductivity change in a given element ∆τ [24]. In the present study, the conductivity change was assumed to be uniform within the volume element. Hence, the general expression of ∆Z_x_, transforms into (2):

(2) $$\Delta Z_{x}\approx -\Delta \sigma \int_{\left(\Delta \tau \right)}\frac{{\vec{E}}_{volt}}{I_{volt}}\frac{\vec{E}{'}_{curr}}{I_{curr}}d\tau$$

E_volt_/I_volt_ is the field of the voltage electrode in the initial medium. E'_curr_/I_curr_ is the lead field of the current electrodes in the medium perturbed by ∆σ in element ∆τ. If the element volume and the conductivity change are small enough, it is convenient to consider that E'_curr_ is approximately equal to the lead field of the current electrodes in the non-perturbed medium. Using Ohm's law, the voltage changes due to the conductivity change is given by (3), where I_S_ denotes the measurement of the current injected across the source electrodes and σ the initial conductivity value within the pixel:

(3) $$\begin{array}{l} \delta u=-\Delta Z_{x}\times I_{S}\approx \left(I_{S}/\sigma \right)\times \left(\Delta \sigma /\sigma \right)\Lambda_{\tau }\\ \text{with }\Lambda_{\tau }\;=-\int_{\Delta \tau }\frac{J_{volt}}{I_{volt}}\frac{J_{curr}}{I_{curr}}d\tau \end{array}$$

Sensitivity Λ_τ_ is expressed in m^-1^ in general (3D) and dimensionless in 2D (translationally uniform model). In the latter case, infinitely long lines model the electrodes and the measurement current in (2) is in A/m, so that the dimensions in (2) and (3) remain consistent.

The sensitivity of all pixels and all electrode patterns used for data acquisition form the sensitivity matrix formed by N_meas_ rows and N_A_×N_L_ columns. The so-called "fan3" electrode pattern has a larger sensitivity domain than the other bipolar drive patterns tested: adjacent, diametric and fan4 [25].

**Figure 3:**
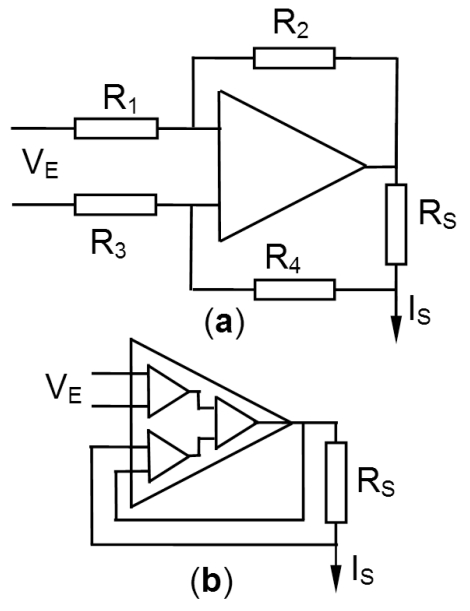
Construction of fan3 pattern. The current injection circuit, (S), is successively connected to the pairs of source electrodes. Fan3 comprises two groups of patterns. The first group uses electrode #3 successively associated to electrodes #9 to #15 (7 patterns). The second group (not shown in this figure for clarity) consists of symmetrical patterns versus the axis of symmetry of the voltage electrodes. In this group electrode #14 is successively associated to electrodes #2 to #8. One pattern {3, 14} is its own image in the symmetry, so that fan3 consists of 13 measurement patterns.

The adjacent drive pattern has widely been used in EIT. It consists of 4-electrode patterns where both voltage and current electrode pairs consist of adjacent electrodes. Fan3 consists of 4-electrode patterns where the voltage electrodes are adjacent and source electrodes are of variable spacing (Figure 3). In fan3, the two source electrodes are separated by the symmetry axis of the voltage electrodes. The sixteen angular replications of these 13 patterns yields N_meas_ = 208 measurement patterns. Similar definition applies to fan4 to fan8 patterns. However fan3 was found to give the largest sensitivity range, so that the other fanX patterns were ignored in this study.

## NOISE

### Noise condition

This section describes the sources of noise and presents the derivation of an equation for the measurement current. In the following, the term "signal" denotes the change in the voltage difference across the sensing electrodes in measuring the perturbed medium and the initial medium (δu in (3)).

The correct measurement of a conductivity change, ∆σ, in an element implies that the sensitivity of this element is above the noise level. It was assumed in this study that the minimum conductivity change to be measured, denoted |? ∆σ/σ |_min_, was the same for all pixels of the mesh. In all drive patterns, certain pixels have low sensitivity values due to either their distance to the probe or the local orthogonality of the lead fields. The contributions of such elements remain under the noise level for any realistic value of the measurement current. Hence, the noise condition used in this study was the following: any pixel of the mesh is sensed by at least one of the N_E_ angular replications of any basic electrode pattern. This condition is really a minimal condition, for, if it were not satisfied, certain measurements would ignore certain pixels. This condition means that for any basic pattern, the contributions of at least N_A_/N_E_ pixels per layer are above noise level. Hence, with a 16-electrode probe and the 64 angular sectors, the relevant parameter is then the fourth largest sensitivity magnitude in each layer of the mesh.

### Noise sources

Three types of noise have been considered in the present study: electrode noise, electronic noise and current noise. Electrode noise's origin is electrochemical. In the frequency range of impedance measurements (f>1 kHz), the general expression for such noise is similar to that of thermal noise [26-27]:

(4) $$\begin{array}{l} {\bar{\varepsilon }}_{el}=\sqrt{4kTBℜ}=Nf_{el}\sqrt{ℜ}\sqrt{\text{B}}\\ {\text{with Nf}}_{\text{el}}=0.123nV{\left(\sqrt{\Omega \text{Hz}}\right)}^{-1}\text{ at 20}{}^{\circ}\text{C} \end{array}$$

where k is Boltzmann's constant, T absolute temperature (°K) and Â the real part of the interface impedance and B system's bandwidth (Hz).

For the quantification of the noise generated in the current injection circuit, the injecting circuit was considered as a voltage-to-current converter of transadmittance G/R_S_(S/m), where G is the voltage gain of the circuit and R_s_ the resistance across which the feedback voltage is measured. Figure 3 shows two circuits commonly used for voltage-to-current conversion: Howland's circuit and differencing amplifier.

The output current is I_S_=V_E_×G/R_S_. G denotes the closed loop voltage gain of the amplifier. Depending on the configuration of the circuit, the noise gain, G_noise_, can be different from G. Let Nf_I_ be the noise figure (V/√Hz) at the input of the circuit. The injected noise current, of rms value denoted i_noise_, is then given by (5) where B is the bandwidth (Hz) of the system:

(5) $$I_{noise}=G_{noise}Nf_{I}\sqrt{B}/R_{S}$$

This noise current produces an error voltage across the measured impedance Z_x_. Considering that the measured impedance is the quotient of the geometry factor g_x_ to the mean conductivity of the medium, σ_m_, the noise voltage due to the current source is given by (6):

(6) $${\bar{\varepsilon }}_{I}\approx G_{noise}Nf_{I}\left|g_{x}\right|\sqrt{B}/\sigma_{m}R_{S}$$

The value of g_x_ can be either calculated for each element and electrode pattern used by means of an appropriate model or derived from experimental measurements of impedance Z_x_, and medium's mean conductivity σ_m_. Table 1 shows the values of g_x_ calculated using the described 2D model and measured *in vitro*. The difference between measured and calculated values has been attributed to the dispersion of current streamlines at the extremities of finite electrodes [9] of length equal to the diameter of the probe as described in section "Experimental setup".

**Table 1:** Limit values of the 2D-calculated and experimental geometry factors for fan3 and adjacent drive patterns

	**fan3 calculated**	**fan3 measured**	**adjacent calculated**	**adjacent measured**
g_max_	0.333	0.254	0.0958	0.151
g_min_	0.125	0.123	0.0124	0.00945

The rms value of the amplifier input related noise voltage is given by (7):

(7) $${\bar{\varepsilon }}_{v}=Nf_{V}\sqrt{B}$$

The noise figure Nf_V_ is given in technical data sheets of operational amplifiers. Finally, using (4), (6) and (7), the total noise rms voltage superimposed to the signal ∆v_x_ is given by:

(8) $${\bar{\varepsilon }}_{T}=\sqrt{2}{\left({\bar{\varepsilon }}_{el}^{2}+{\bar{\varepsilon }}_{I}^{2}+{\bar{\varepsilon }}_{V}^{2}\right)}^{1/2}\sqrt{B}$$

The coefficient √2 accounts for the fact that the "signal" is the difference between two measurements.

### Numerical application

The noise figure of the voltage amplifier is equal to 13nV/√Hz, which corresponds to standard op-amps usable in EIE. The contact impedance of one electrode of this probe in tap water of conductivity 0.039 S/m was 400 Ω? at the used measurement frequency of 8 kHz [8]. In a urethral probe, the electrode surface would be reduced by a factor of about 25 or less with respect to the mock-up probe used in bench experiments. The interface impedance would then become about 10 k Ω in tap water. However, tissue conductivity is in general higher than that of tap water, so that the interface impedance will presumably be lower in tissue than in tap water. In the absence of literature data for urethral wall, data for blood vessel and prostate were considered instead. From the data published on the website of the Institute for Applied Physics "Nello Carrara" [28], the magnitude of the calculated prostate admittivity (?σ*= σ_0_+jωɛɛ_0_) varies from about 0.43 S/m to 0.58 S/m in the prostate and from 0.28 to 0.33 for blood vessel in the range 10 kHz - 1 MHz. Furthermore, the possible presence of urine, wetting urethra wall, would presumably tend to decrease the contact impedance.

The presence of urine of higher conductivity than the surrounding tissue, can potentially affect the measurements. Besides the reduction of electrode contact impedance due to the wet urethral wall, the presence of urine could also cause adjacent electrodes to short circuit. It may be expected that the amount of urine present during the measurements would be limited by the preliminary draining of the urethra and the temporary obstruction of the lumen by the tip of probe. The shorting impedance would depend on the thickness of the conductive layer forming between the probe surface and the urethral wall. A possible protection measure would be to give electrode edges a slightly salient profile to locally increase the pressure to constrict or divide the conductive layer. This issue can only be solved by a practical measurement *in situ*.

Conductivity values for urine range from 2.5 S/m to 4.5 S/m [29, 30]. In this study, in the worst case the real part of the interface impedance of two electrodes in series was finally maximised by 2500 Ω. The corresponding electrode noise figure, Nf_el_, is then about 6 nV√Hz. The noise figure due to the current injecting circuit can be derived from (6). With G_noise_ = 2, Nf_I_ = 13nV/√Hz, |g_x_| = 0.333 (maximal value in Table 1), R_S_ = 1 kΩ and σ_m_ = 1 S/m, the noise figure due to the current source is about 0.009nV/√Hz, which is negligible compared with the other sources of noise. The total noise figure in this numerical example is then about 14.3nV/√Hz.

### Noise condition and measurement of current equation

The condition for the correct measurement of the contribution of an element is that the contribution, δu, of this element is above noise level. Assuming that the distribution of noise amplitude is Gaussian, one may take 3.09×N_f_ as arbitrary noise threshold, with the risk of 0.002 for the instantaneous noise voltage, which is outside the interval ±3.09×N_f_. Using (4), (5), (6) in (8) and grouping the terms corresponding to the probe, the instrumentation and the medium finally gives the expression of the rms value of the injected current satisfying the above noise condition:

(9) $$\begin{array}{c} I_{S}\geq \left(3.09\sqrt{2}/\left|\Lambda_{x}\right|\right)\times \\ {\left({\left(Nf_{el}\sqrt{ℜ}\right)}^{2}+{\left(G_{noise}Nf_{I}g_{x}/\sigma_{m}R_{S}\right)}^{2}+Nf_{V}^{2}\right)}^{1/2}\times \\ \sqrt{B}\left(\sigma_{m}/{\left|\Delta \sigma /\sigma \right|}_{\min}\right) \end{array}$$

The mean conductivity of the medium, σ_m_, depends only on the explored medium, the conductivity change σ/σ on the observed phenomena (tumour, treatment).

## DESIGN PARAMETERS

The purpose of this section is to determine the realistic design values for incorporation into (9) of the mean conductivity, σ_m_, and minimal conductivity change |∆σ/σ|_min_. The mean conductivity value determines the magnitude of the measured impedance Z_x_. and consequently that of a pixel's contribution, δu. For the prostate, Dawson [31] reports a value of 0.4 S/m for studies at 60 Hz. This value is close to the static conductivity σ_0_ of the prostate given by the IFAC internet resource [28]. The conductivity values calculated in section "Numerical application" from this resource for normal prostate tissue (0.43 to 0.58) suggested 0.5 S/m as design value of σ_m_ in (9).

The design value of |∆σ/σ|_min_ corresponds to the smaller change due to either the presence of cancer tissue or the treatment of a tissue by therapeutic ultrasound. Several sources of data enable the estimation of ∆σ/σ in presence of cancerous tissue. Blad [32] has proposed a general conductivity ratio between normal tissue and cancer tissue of about 0.69 (|∆σ/σ| = 0.31). Conductivity ratios of 0.72 and 0.78 at 16 kHz and 125 kHz, respectively, were observed between carcinoma and glandular tissue in excised breast tissue samples [17, 18].

Dunning tumour in a Copenhagen rat is a commonly used model for human prostate cancer [33]. The admittance of growing AT2 Dunning tumours was monitored during 21 days [34]. The conductivity was estimated by modelling the tumour with a cylindrical segment of length equal to its diameter with four equally spaced electrodes. This yielded a rough estimate of tumour conductivity of about 0.2 S/m at 9 kHz and 0.4 S/m at 1 MHz. Using the figures and the values for reference prostate tissue of section "Numerical application", the coarse estimates of conductivity ratios are about 0.47 and 0.69 at the two considered frequencies.

Lee [19] carried out impedance measurements at 100 kHz, 1 MHz, 2 MHz and 4 MHz in prostates *ex-vivo* using bio-impedance needles. The conductivity was smaller in cancer tissue than in prostate tissue at 100 kHz, 1 MHz and 2 MHz with conductivity ratios of 0.86, 0.92 at and 0.8, respectively.

Smith, by measuring eddy currents using a magnetic coil at 2.14 MHz, compared the conductivity values in Dunning tumours from G, AT2 and AT3 lines [20]. The conductivity was lower in AT2 and AT3 tumours (0.22 and 0.24 S/m) and G line (0.33 S/m) than in control tissue (0.35 S/m). From the above data, the value of 10% was taken as representing the minimal change |∆σ/σ|_min_ resulting from the presence of prostate cancer.

**Figure 4:**
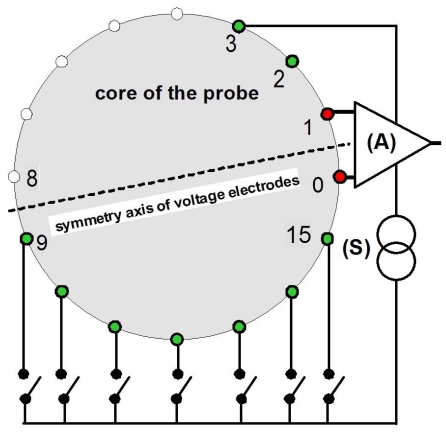
Two circuits for voltage-to-current conversion commonly used in impedance measurement. In both cases, the output current, IS, is equal to GVE/RS. In the Howland circuit (a), the voltage gain is G=R2/R1=R4/R3 and the noise gain is G+1. Using a difference amplifier (b), the voltage gain and noise gain are both equal to unity.

The energy deposited in tissue by therapeutic ultrasound produces the irreversible necrosis of the tissue. *In vitro* experiments showed noticeable changes in a tissue's impedance. Changes larger than 20% were observed in tissue samples exposed *in vitro* to high energy ultrasound [21] and muscle tissue samples [22]. As these values were larger than the value for cancer tissue, the latter (10%) was finally taken as design value of |∆σ/σ|_min_ for incorporation into (9). Figure 4 shows the plot of the measurement current satisfying the noise condition (9) with the value of section “Numerical application” and |∆σ/σ|_min_ = 0.1. In this plot, the parameter varying with distance is Λ_x_, the fourth largest sensitivity value calculated for each layer of the mesh described in Figure 2.

## SIMULATION

This section describes the simulation of an EIE application using calculated and experimental data. The conductivity ratio (σ/σ_0_) was set to 1.1 for the simulation of biological conditions and to zero to simulate the plastic rods used in bench experiments. The model described below was used for the simulation of two conductivity perturbations, of radius 0.2 and 0.3 and centred on the Oy axis at (0,2) and (0,3), respectively. The experimental data were collected using the bench model described in section “Experimental setup”.

### Software model

The signal, the change in the measured potential difference across a pair of sensing electrodes, was calculated using the 2D software model developed for the project [35]. In this model, infinitely long lines represent the electrodes and all quantities are assumed constant by translation along one direction. This models yields analytical equations for electric field and potential. The conductivity perturbations were assumed to be infinitely long cylinders parallel to the axis of the probe and projecting on the calculation plane as circular disks of conductivity σ=σ_0_+∆σ. The voltage change at the sensing electrodes was calculated using the image theory [36] considering the series of images of the initial source electrodes in the perturbing cylinder and in the probe. This forms sequences of sources with rapid convergence of potential and electric field.

The addition of noise to calculated data was achieved according to (11):

(11) $$\begin{array}{c} \Delta u_{n}(k)=\left(I_{meas}/\sigma_{m}\right)\times \\ \left(\Delta u_{calc}(k)+N_{L}\times {\left\|\Delta u_{calc}\right\|}_{2}\times X(k)\right) \end{array}$$

||∆u||_2_ is the 2-norm of the data vector calculated for I_s_/σ_m_ equal to unity. N_L_ denotes the noise level (dimensionless) and X a normal Gaussian variable. Hence, assuming that the variance of sample X_k_ is equal to the variance of the distribution, the relation between total noise, ɛ_T_ and added noise is given by (12):

(12) $$N_{L}=\varepsilon_{T}\sigma_{m}/\left(I_{meas}{\left\|\Delta u_{calc}\right\|}_{2}\right)$$

### Inverse problem

In the linear approximation, the measured potential changes are assumed proportional to the conductivity changes. The images were reconstructed solving the normal matrix equation:

(13) $$\text{A.}\Delta \sigma =\text{b with A = }\Lambda^{\text{T}}.\Lambda \text{ and b = }\Lambda^{\text{T}}\Delta \text{u}$$

**Figure 5:**
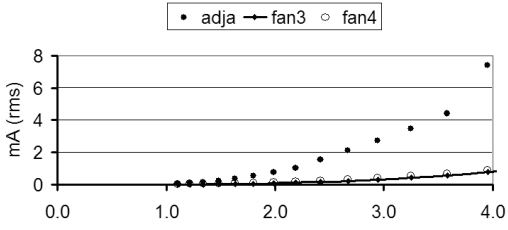
Calculated measurement current (mA rms) needed to satisfy the noise condition of (9) with the mesh of Figure 2 under the conditions of the numerical example, section "Numerical application and |∆σ/σ| = 0.1 (Section 4). The horizontal axis is the normalised distance (R_probe_ = 1) from the origin to the centre of the pixel.

∆**σ** is the unknown vector of conductivity changes, ∆**u** is the vector of the measured potential changes and **Λ** the sensitivity matrix. The sensitivity matrix is ill-conditioned. The plot of singular values shows that the maximal rank of this matrix is 104 with 16 electrodes (Figure 5), which corresponds to the number of linearly independent measurements. Fan3 and fan4 show very similar sets of singular values. Experimental measurements confirmed that these two types of drive have equivalent performances so that fan4 was ignored in the following sections. The largest singular value of adjacent drive is about four times smaller than that of fan3.

### Reconstruction method

The equation was solved as an optimisation problem using Tikhonov's regularisation method, searching for a vector b minimising the functional F defined by:

(14) $$\begin{array}{l} \text{ F}={\left\|A\Delta \sigma -b\right\|}_{2}^{2}+\lambda^{2}{\left\|b\right\|}_{2}^{2}\\ \text{with }A\;=\Lambda^{T}\Lambda \text{ and }b=\Lambda^{T}\Delta u \end{array}$$

Symbol ||**w**||_p_ denote the p-norm of a vector **w** and λ is the regularisation parameter. The optimal value of the regularisation parameter λ was determined automatically for each data set using the "L-curve" procedure. The lower limit of λ (10^-15^) was determined by successive trials using simulated data. For this value, the images reconstructed from simulated noiseless data could not be distinguished from reconstruction noise. The upper limit (10^-4^) corresponded to clearly excessive image smoothing producing lobes spreading over the entire image. In practice, the values found by the automatic L-curve procedure ranged roughly from 10^-9^ to 10^-12^ for noiseless simulated data and from 10^-6^ to 10^-8^ for experimental data. Regularised matrices were pre-calculated using three values of λ per decade. Image reconstruction therefore consisted of matrix-vector products and calculation of the radius of curvature of the L-curve. The calculation was carried out using the circular mesh of Figure 2 using specific software written in Borland Delphi. The cartesian mesh for 3D plots of Figures 8 to 10 were obtained by linear interpolation. With a 1.6 MHz laptop PC computer the image was available in about 20 seconds.

## EXPERIMENTAL SETUP

The experimental data set were collected using a bench system that comprised a 16-electrode mock-up probe, 50 mm in diameter, immersed in a tank filled with tap water modelling a uniform conductivity medium and the purpose-built experimental instrumentation [9]. The electrodes, made of brass, formed 50×2 mm^2^ conducting stripes regularly spaced around the probe. The measurement frequency was 8 kHz [8]. This frequency was chosen for bench experiments as it ensured satisfactory compromise between the increase of electrode-medium interface impedance at low frequency and the onset of error due to stray capacitance with increasing measurement frequency. The magnitude of the measurement current was 1mA pp. The contact impedance was about 400 ohms per electrode in tap water.

Tap water has been widely used in EIT as a uniform conductivity model, especially for feasibility studies and test of instrumentation, even though it does not have the same electric and dielectric properties as human body tissues. As a matter of fact, there is no really satisfactory model of the conductivity of cellular medium. The conductivity of tap water was 0.039 S/m in this study. Furthermore, the use of a liquid model makes it easier to place conductivity perturbations in the medium.

**Figure 6:**
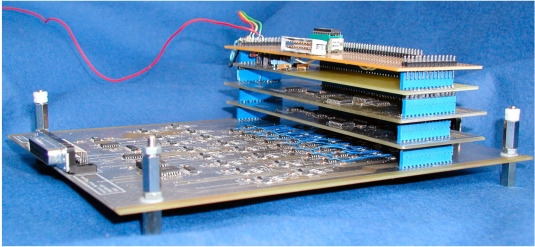
Present version of the front end. The largest board comprises 16 electrode buffers, 16 differential voltage amplifiers. The other boards are two multiplexers boards for the selection source and sensing electrode pairs, the current source with the current measuring circuits and the control logic. The electrodes are connected to a 16-line linking the four stacked printed boards. Stacking boards enables flexibility for successive versions of the hardware system.

The adaptable front-end system (Figure 6) comprised a controlled amplitude current source based on the circuit of Figure 3a, 16 input differential voltage amplifiers, 4 multiplexers for the 16 to 1 selection of electrodes. In this study, the demodulator produced a DC signal proportional to the magnitude of the measured voltage. This voltage was digitized with a resolution equivalent to 17 bits. This was achieved by means of a two-stage system reducing the DC offset of the signal using a D/A converter and sampling the amplified residual with a 12-bit A/D converter.

**Figure 7:**
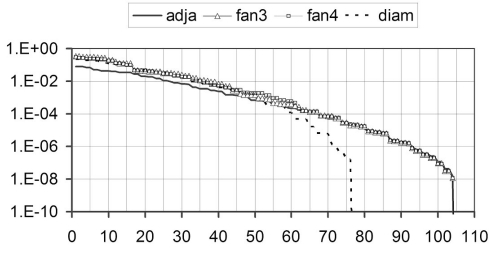
Singular values of the sensitivity matrices for diametric, adjacent, fan3 and fan4 bipolar drive patterns. The rank was 76 for diametric drive and 104 for all other drive patterns.

## RESULTS

### Images reconstructed from noiseless calculated data

**Figure 8:**
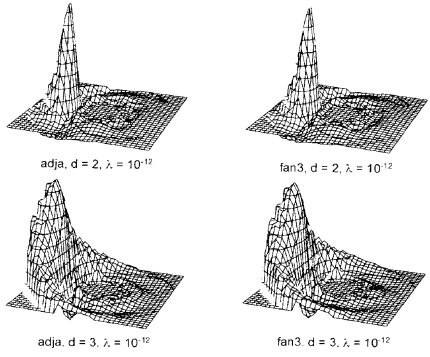
Images reconstructed from calculated noiseless data for adjacent and fan3 drive patterns. The simulated conductivity perturbations (∆σ/σ=0.1, radius r = 0.2) were centred at d = 2 (top row) and d = 3 (bottom row). Regularisation coefficient was λ = 10^-12^ for all four images.

Figure 8 shows the images reconstructed according to (14). The drive patterns fan3 and adjacent give similar images. For both types of drive, the resolution is better near the probe and deteriorates for increasing distance from the probe.

### Images reconstructed from calculated data with added noise

**Figure 9:**
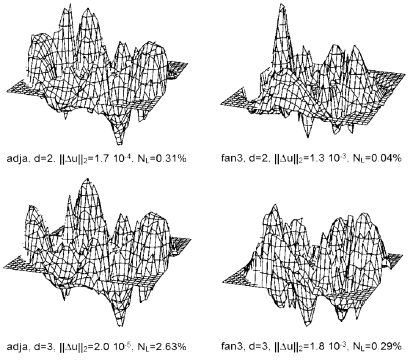
Images reconstructed using the same calculated data in Figure 8 with the addition of a constant gaussian noise voltage representing electrode and amplifier noise. The resulting noise level N_L_ varied according to the sensitivity of each drive pattern and signal magnitude. The regularisation factor was λ = 3.16×10^-7^ for all images.

Gaussian noise was added to the calculated data according to (11). The simulated real part of electrode contact impedance was 400 Ω, simulating the value measured *in vitro*. The amplifier's input related noise figure was taken equal to 13 nV/√Hz. Current noise was ignored. The resulting total noise figure was 13.2 nV/√Hz. The bandwidth was B = 208. Figure 9 shows the corresponding images. The conductivity perturbation does not distinguish from noise artefact, excepted for fan3 drive pattern at distance d = 2. This figure illustrates the influence of noise and the different susceptibility to noise of adjacent and fan3 drive patterns.

### Images reconstructed from experimental data

**Figure 10:**
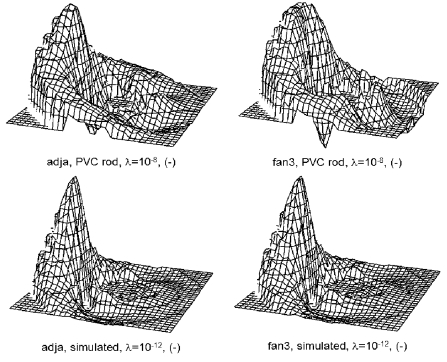
Images from experimental data obtained with a PVC cylindrical rod, 16 mm in diameter (normalised radius r = 0.32) and located at distance d = 3 (top row) compared to the images from simulated noiseless data of the same perturbation (bottom row). In this figure, the sign of the reconstructed values has been inversed to display the upward negative perturbations of conductivity.

The experimental data sets were collected using the set-up described above. The perturbation used in this study was an insulating PVC, 16 mm in diameter (normalised radius of 0.32) and located at distance d = 3 from the axis of the probe. Figure 10 shows the images reconstructed from the *in vitro* data and from calculated noiseless data modelling the same perturbation.

## DISCUSSION

The above data and design parameters confirm that EIE is particularly sensitive to noise. This is mainly due to the rapid decrease of sensitivity with increasing distance from the probe due to the simultaneous reduction of the lead fields of current and voltage electrodes. The results obtained in this study are compatible with prostate size. The noise of the current injection circuit is negligible compared to the other sources of noise. The predominant source of noise is the input related noise of the voltage amplifier. The second largest source of noise is electrode noise. This gives particular importance to electrode technology in a miniaturised probe.

For tissue characterisation, there is no particular need for high data acquisition speed. The parameter bandwidth (B) in (9) accounts for measurement time. The above numerical examples were based on the rate of 1 frame per second with 208 values per frame. The increase of the total acquisition time by, for instance, a factor of 16, would be practically acceptable and would increase by 12dB the signal-to-noise ratio. With the limit fall-off in 1/d^4^ of the sensitivity this would theoretically increase by two the sensitivity range, reduce the measurement current or improve the quality of the reconstructed images. One possible technique would be the averaging of 16 successive images. The use of a series of images would also enable the detection of transient artefact during data acquisition.

The limit size for the detection of a tumour depends on the conductivity of the medium, the conductivity contrast of the tumour, the sensitivity distribution of the used drive pattern, the measurement noise and the magnitude of the applied current. There is therefore no unique answer to the question of limit size for detection. However, the plots in Figure 4 enable the calculation of estimates under the conditions of example numerical application. These plots correspond to the limit measurement conditions for the pixels of the mesh of Figure 2. Straightforward considerations based on equation 9 yield a value for given current magnitude and tumour location. Table 2 shows the values obtained with a conductivity ratio ∆σ/σ = 10% for 1mA and 10mA currents at the distance of three times the radius of the probe, assumed as typical range for EIE measurements. For extrapolation of the figures of Table 2 to conductivity changes than 0.1, the conductivity ratio q=∆σ/σ should be replaced with the quantity 2q/(q+2) ratio in equation 9 to account for non-linearity [35].

**Table 2:** Limit diameter of a conductivity perturbation (∆σ/σ = 10%) located at three times the radius of a probe under the example measurement conditions. Figures in percent are relative to the diameter of the probe. Figures in mm are for a probe 7 mm in diameter

	adjacent	fan3	adjacent Ф = 7 mm	fan3 Ф = 7 mm
1 mA	25%	8.4%	1.8 mm	0.6 mm
10 mA	7.9%	2.7%	0.6 mm	0.2 mm

The two selected bipolar drive patterns were adjacent and fan3. Adjacent drive has widely been used in EIT due to its minimal number of measurements and its full ranked sensitivity matrix. Fan3 was selected in previous studies for its sensitivity range that was found larger than that of the other tested patterns [9,26]. The required number of measurements in fan3 is twice that of adjacent drive and the sensitivity matrix is not full rank. However, the additional linearly dependent data can be seen as an averaging improving signal-to-noise ratio in the same way as the full set of 208 measurements used in EIT to compensate for reciprocity error. The reconstructed images indicate that adjacent and fan3 give images of similar quality in absence of noise. Adjacent drive requires a four time larger measurement current for a given signal-to-noise ratio (Figure 4). This can be compensated by sufficient current magnitude and measurement time. Under these conditions, the above noise equation shows that both types of drive can be used. Preference would then be given to adjacent drive due to its better matrix conditioning. In practice, however, the use of small size electrodes could potentially limit the actual magnitude of the applied current below the maximal limit of 10 mA rms. Furthermore, the impedance of electrodes and the output swing of the current source can also limit the magnitude of the measurement current. Table 2 shows that fan3 enable the detection of larger tumour than fan3 for given measurement conditions. This argument may be decisive in selecting the drive pattern to be implemented. In any case, electrode technology will be crucial in the design of probes and that, finally, EIE seems more appropriate to tissue characterisation than to high speed imaging.

## CONCLUSION

The simulation of operating conditions enabled the quantification of the magnitude of the measurement current ensuring appropriate signal-noise ratio. Measurement current of about 1 mA satisfying the noise condition derived in this study is feasible in practice. This study also showed that fan3 and adjacent drive patterns give similar results with noiseless data, but that adjacent drive requires significantly higher measurement current than fan3. The equation derived in this study enables the specification of the hardware system given the operating condition of a given application.
